# The generation of centripetal force when walking in a circle: insight from the distribution of ground reaction forces recorded by plantar insoles

**DOI:** 10.1186/1743-0003-12-4

**Published:** 2015-01-09

**Authors:** Anna Maria Turcato, Marco Godi, Andrea Giordano, Marco Schieppati, Antonio Nardone

**Affiliations:** Posture and Movement Laboratory, Division of Physical Medicine and Rehabilitation, Scientific Institute of Veruno, Fondazione Salvatore Maugeri (IRCCS), Veruno, NO Italy; Department of Translational Medicine, University of Eastern Piedmont, Novara, Italy; Unit of Bioengineering, Scientific Institute of Veruno, Fondazione Salvatore Maugeri (IRCCS), Veruno, NO Italy; Centro Studi Attività Motorie (CSAM), Scientific Institute of Pavia, Fondazione Salvatore Maugeri (IRCCS), Pavia, Italy; Department of Public Health, Experimental and Forensic Medicine, University of Pavia, Pavia, Italy

**Keywords:** Plantar pressure, Ground reaction force, Gait, Curved trajectories, Trunk inclination

## Abstract

**Background:**

Turning involves complex reorientation of the body and is accompanied by asymmetric motion of the lower limbs. We investigated the distribution of the forces under the two feet, and its relation to the trajectory features and body medio-lateral displacement during curved walking.

**Methods:**

Twenty-six healthy young participants walked under three different randomized conditions: in a straight line (LIN), in a circular clockwise path and in a circular counter-clockwise path. Both feet were instrumented with Pedar-X insoles. An accelerometer was fixed to the trunk to measure the medio-lateral inclination of the body. We analyzed walking speed, stance duration as a percent of gait cycle (%GC), the vertical component of the ground reaction force (vGRF) of both feet during the entire stance, and trunk inclination.

**Results:**

Gait speed was faster during LIN than curved walking, but not affected by the direction of the curved trajectory. Trunk inclination was negligible during LIN, while the trunk was inclined toward the center of the path during curved trajectories. Stance duration of LIN foot and foot inside the curved trajectory (Foot-In) was longer than for foot outside the trajectory (Foot-Out). vGRF at heel strike was larger in LIN than in curved walking. At mid-stance, vGRF for both Foot-In and Foot-Out was higher than for LIN foot. At toe off, vGRF for both Foot-In and Foot-Out was lower than for LIN foot; in addition, Foot-In had lower vGRF than Foot-Out. During curved walking, a greater loading of the lateral heel occurred for Foot-Out than Foot-In and LIN foot. On the contrary, a smaller lateral loading of the heel was found for Foot-In than LIN foot. At the metatarsal heads, an opposite behaviour was seen, since lateral loading decreased for Foot-Out and increased for Foot-In.

**Conclusions:**

The lower gait speed during curved walking is shaped by the control of trunk inclination and the production of asymmetric loading of heel and metatarsal heads, hence by the different contribution of the feet in producing the body inclination towards the centre of the trajectory.

## Background

Human walking has been extensively studied from both the kinematic and kinetic perspectives [1–3]. Most published studies deal with walking along straight paths. Few papers focus on walking along non-linear, e.g. curved, trajectories. Under the curved-walking condition, the central nervous system organizes the movement considering not only the propulsion required but also the equilibrium constraints connected to body rotation. Turning involves complex reorientation of the head, trunk, pelvis and feet [4–8], and is accompanied by adjustments of body orientation (such as trunk inclination to the inner part of the trajectory) to counteract the centrifugal acceleration acting on the walking body, and by asymmetric motion of the lower limbs, whereby the leg on the inside of the trajectory travels a shorter pathway than that on the outside [4, 9]. Not unexpectedly, recent studies requiring subjects to travel both linear and circular pathways have detected abnormalities in patients with neurological disorders. Both Parkinson’s disease [10] and stroke patients [11] show walking difficulties, more evident during circular rather than linear trajectories. Therefore, given the fundamental and clinical relevance of curved walking, the present investigation was carried out to investigate foot action during curved walking, in the hypothesis that the distribution of the vertical forces under the feet can help in producing the coordinated motion of the turning body.

Knowledge about the spatio-temporal pattern of distribution of the ground reaction forces (GRF) when walking in a circle may be useful for assessing the control of medio-lateral equilibrium and the way subjects and patients accomplish the task. It could help in detecting changes due to diseases of the central [11–13] or peripheral nervous system [14], and in estimating the evolution of the walking disorder or the potential advantage of gait rehabilitation [15, 16]. Plantar pressure analysis from different points of the foot sole [17] has a high degree of reliability [18, 19], and can be useful and appropriate for such assessment.

We hypothesised that: 1) the distribution of the forces beneath the sole differs between linear and curved trajectories, owing to the different kinematics of the two feet during curved walking; 2) the force distribution during curved walking differs between the inner and outer foot, owing to the need to exert different GRFs in order to produce the centripetal acceleration; and 3) the placement of the point of application of the forces under the foot sole should help create or modify the torques acting in the frontal plane, so as to match the body’s internal inclination during curved walking.

We analyzed the GRF collected by insole devices, since it gives an overall view of the time-course of the vertical forces and its peaks [6, 20–22]. The combination of spatial distribution, time-course, and peak values of GRF in selected parts of the feet provides detailed information about the features of the forces acting on the body during linear and curvilinear paths, and gives insight into the generation of the centripetal force when walking in circle. Preliminary results have been recently presented [6, 23].

## Methods

### Subjects

Twenty-six healthy participants (16 women, 10 men), aged (mean ± standard error, SE) 25.1 ± 0.5 years, range 21–35, mean body weight 63.0 ± 0.8 kg, mean height 1.7 ± 0.2 m, were recruited. No subject had a history of neurological diseases. All were free from ankle or foot pathology or other impairments that could contribute to postural instability or movement dysfunction. Exclusion criteria were major trauma in the last six months or lower limb surgery at any time previously. The study was approved by the local Research Ethics Committee and all subjects gave their informed consent.

### Procedure

Subjects walked under three different conditions, in random order: linear walking (LIN) and curved walking clockwise (CW) and counter-clockwise (CCW), at self-selected speed. The circle path (1.2 m radius) was drawn with a tape stuck on the floor. Subjects executed two 20 m trials for each trajectory, making a total of 6 trials. Before data acquisition, each subject performed two short practice trials for each condition to familiarize themselves with the instrumentation and task. They were instructed to walk looking forward, head erect, and not to focus on the tape. Walking time was monitored using photocells. For the LIN trajectory, photocells were placed at the beginning and at the end of a 20 m pathway; for the curved trajectories, at the beginning of the first lap and at the end of the third lap, corresponding to an overall path of 20 m. For each subject, we collected 50 steps for each condition. The first and last two steps of each trial were excluded from data acquisition, because changes in spatial-temporal variables (albeit minimal [24]) can occur at initiation and termination of gait [25, 26]. The entire session lasted about one hour.

### Data collection and treatment

Walking speed was determined from the time taken to travel a 20 m pathway for both linear and curved trajectories. Stance duration was the time interval between the heel strike and toe off of the same leg. To allow for more ready comparison between straight line walking and turning, the percentage of the total gait-cycle duration (%GC) was computed as the time-interval between two successive heel strikes of one leg. In all subjects, both feet were instrumented with insoles. Subjects wore no socks. Insoles (Pedar-X system, Novel, Germany) and shoes (Superga 2750 model, Italy) corresponding to the individual’s foot size were chosen. Insoles were placed inside the shoes and connected to the Pedar box. At the beginning of the session, the insoles were calibrated using the proprietary calibration device according to the manufacturer’s manual. Data were sampled at 50 Hz. The Pedar-X system used in this study has been previously shown to have good reliability for both linear and curved trajectories [19].

The system produces, for each sample, a force value measured as the sum of the forces registered by the active sensors from each insole: this force value was used as the vertical component of the ground reaction forces (vGRF). The time course of the vGRF was stored for each trial and subsequently analysed using proprietary software.

The insole was also divided into eight anatomical foot regions: medial and lateral heel, medial and lateral arch, I metatarsal head, II-V metatarsal heads, hallux, and lateral toes. This was done in order to describe the different contribution of each region to the total vGRF averaged across the 50 steps [18]. vGRF was normalized to body weight (%BW = vGRF /BW*100) to reduce inter-individual variability [27, 28].

We also measured trunk inclination during linear and curved trajectories by means of a tri-axial accelerometer (MicroStrain G-LINK Wireless Accelerometer System, ± 2 g range). This was placed in a pocket fastened by an elastic belt to the sternum, in the midline and midway along its length. Data were transferred wirelessly to a PC for off-line analysis. Acceleration data were sampled at 32 Hz; inclination was estimated using a low-pass FIR filter (finite impulse response filter, cut-off frequency = 0.3 Hz, 50th order/51-tap) in order to obtain the gravitational component of the medio-lateral acceleration, followed by trigonometric transformation [29].

### Statistical analysis

We used parametric statistics, since the variables to be compared (vGRF, stance duration, walking speed, trunk inclination) followed a normal distribution (p > 0.05, Shapiro-Wilk’s Test) and variances were homogeneous (Levene’s Test, p > 0.05 for all variables). One-way analysis of variance (ANOVA) assessed differences in walking speed under the three walking conditions (LIN, CW, CCW). Two-way ANOVA was performed for vGRF and stance duration of the left and right foot during the three walking conditions. During curved trajectories, meaningful functional comparisons between feet were made according to the position of the foot with respect to the trajectory, i.e. foot on the inside (‘Foot-In’) or on the outside (‘Foot-Out’). Values of vGRF from each foot anatomical region were compared in the three conditions (LIN, Foot-In, Foot-Out) with one-way ANOVA. When ANOVA gave a significant result (p < 0.05), post-hoc analysis was performed with Tukey’s test. CW and CCW have been examined as different conditions. Since the major gait determinants such as speed and cadence were not different between curved trajectories (see Table 1), the plantar vGRF of the feet were collapsed into a Foot-In and a Foot-Out prior to statistical analysis. Data are presented as means ± SE in the figures and the text. The software STATISTICA (StatSoft, version 12.0) was used.

**Table 1:** **Gait variables during different walking trajectories**

Gait variables	Walking trajectories			ANOVA				
	LIN	CCW	CW	F	df	P		Post-hoc
Speed (m/s)	1.59	1.35	1.33	14.9	2,76	**<0.001**	**<0.005**	LIN vs CCW
*SE*	*0.06*	*0.05*	*0.05*				**<0.005**	LIN vs CW
							0.96	CCW vs CW
Cadence (steps/min)	127.1	114.6	114.5	8.2	2,76	**<0.005**	**<0.005**	LIN vs CCW
*SE*	*3.2*	*2.4*	*2.1*				**<0.005**	LIN vs CW
							0.99	CCW vs CW
	**Feet**			**ANOVA**				
	**LIN foot**	**Foot-In**	**Foot-Out**	**F**	**df**	**P**		**Post-hoc**
Stance duration (% GC)	62.8	62.6	61.1	6.5	2, 147	**<0.005**	0.97	LIN vs Foot-In
*SE*	*0.6*	*0.4*	*0.5*				**<0.005**	LIN foot vs Foot-Out
							**<0.05**	Foot-IN vs Foot-Out

SE (standard error), GC (gait cycle), CCW (counterclockwise), CW (clockwise), LIN foot (average of left and right foot during linear trajectory), Foot-In/Out (foot inside/outside the trajectory).

## Results

### Gait variables during linear and curved trajectories

As shown in Table 1, walking speed and cadence were significantly affected by the type of trajectory, both being larger during LIN than curved. On the other hand, during curved-walking, speed and cadence were not affected by the direction of the trajectory.

During LIN, stance duration was similar in the left and right foot (Table 1). During curved walking, stance duration of the Foot-Out was shorter than that of LIN foot and Foot-In, while there was no difference between LIN foot and Foot-In. The changes observed in the above gait variable according to the type of trajectory confirmed previous findings in young [19] and elderly subjects as well as patients [10, 11].

### Ground reaction force during the stance phase

**Figure 1:**
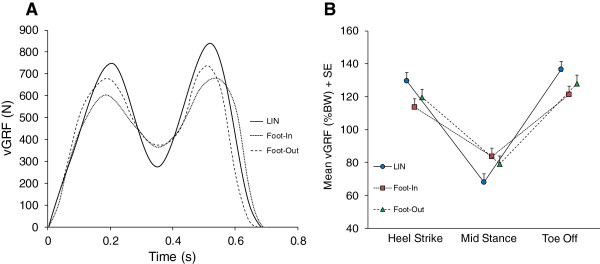
**Vertical component of the ground reaction force (vGRF) during linear and curved trajectories in the outer and inner foot. A**. Profiles of the vertical component of the ground reaction force (vGRF) obtained during stance in the left foot of a representative subject during linear (LIN) walking and in the same foot when it was on the outside (Foot-Out) and inside (Foot-In) of the curved trajectory. **B**. Average of all subjects (+ standard error, SE) of the vGRF normalized to body weight (% BW) measured during the linear trajectory (LIN) as the average of right and left foot values and during the curved trajectory in the inner (Foot-In) and outer foot (Foot-Out). Values are obtained from the peak values at heel strike and toe off, and from the trough value at mid-stance. Average of 50 steps.

Figure 1A shows the vGRF-time profiles during the stance phase in the left foot of a representative subject during LIN and during CW and CCW walking, when the foot was Foot-Out or Foot-In. The profiles are characterized by two peaks separated by a trough: the first peak broadly corresponds to the heel strike and weight acceptance phase (henceforth expressed as heel strike, HS), the trough to the foot mid-stance (MS), and the second peak to terminal stance and toe off (TO). LIN and curved trajectories differently affected the step time profile of vGRF: while this was superimposable for both feet during LIN (not shown), compared to LIN the vGRF profiles of both Foot-In and Foot-Out showed a decrease of the first (HS) and last peak (TO) and an increase of the trough at MS. Further, the trough was relatively larger in Foot-In than Foot-Out (Figure 1B).

**Figure 2:**
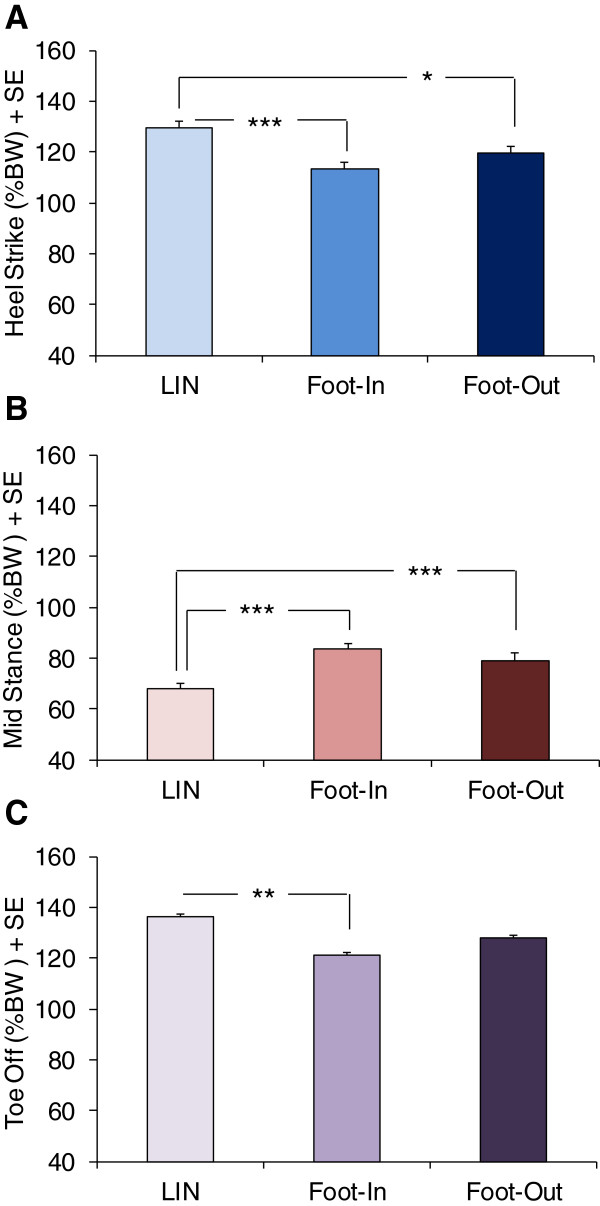
**Average (+ standard error, SE) of the vertical component of the ground reaction force (vGRF) normalized to body weight (% BW) measured during the linear trajectory (LIN) as the average of right and left foot values and during curved trajectory in the inner (Foot-In) and outer foot (Foot-Out).** Values are obtained from the peak values of vGRF at heel strike **(A)** and toe off **(C)**, and from the trough value at mid-stance **(B)**. **A**. At Heel Strike, vGRF of both Foot-In and Foot-Out was lower than that of LIN foot. **B**. At Mid-Stance, both Foot-In and Foot-Out had greater vGRF values than LIN foot. **C**. At Toe Off, Foot-In had smaller vGRF values than LIN foot. *, p < 0.05; **, p < 0.005; ***, p < 0.0005.

At HS, vGRF was 129.6 %BW ± 2.7, 113.6% ± 2.5 and 119.6% ± 2.8 for LIN foot, Foot-In and Foot-Out, respectively (F(2,76) = 9.36; p < 0.0005) (Figure 2A). Post-hoc analysis showed that vGRF of both Foot-In and Foot-Out were lower than that of LIN foot (respectively, p < 0.0005 and p < 0.05). In turn, vGRF was slightly smaller (not significantly so) for Foot-In than Foot-Out (p = 0.25). At MS, vGRF was 62.8%BW ± 2.3, 86.4 ± 2.3 and 80.0 ± 3.1 in LIN foot, Foot-In and Foot-Out, respectively (F(2,76) = 32.0; p < 0.0005) (Figure 2B). Both Foot-In and Foot-Out had greater vGRF values than LIN foot (post-hoc, p < 0.0001). vGRF of Foot-In was marginally higher than that of Foot-Out (p = 0.07). At TO, vGRF was 136.5%BW ± 2.7, 121.3 ± 2.7 and 127.9 ± 2.8 in LIN foot, Foot-In and Foot-Out, respectively (F(2,76) = 7.70; p < 0.005) (Figure 2C). Post-hoc analysis showed that Foot-In had smaller vGRF values than LIN foot (p < 0.005). vGRF was marginally smaller in Foot-Out than LIN foot (p = 0.08).

### Ground reaction force distribution in space

**Figure 3:**
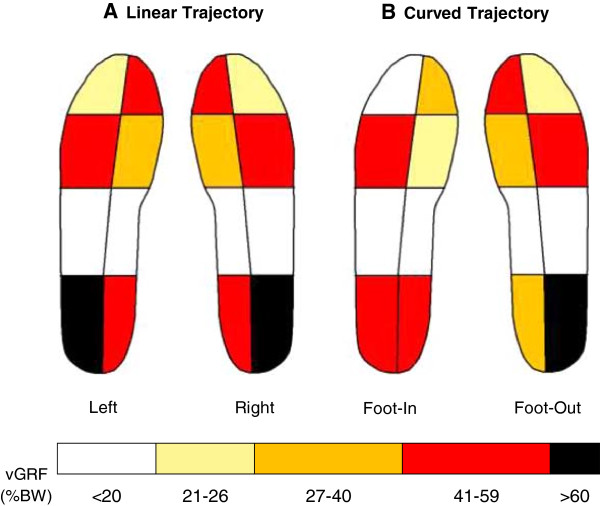
**Estimated ground reaction force normalized to body weight (%BW) measured in each of the eight anatomical regions of the foot during linear trajectory (A) in the right and left foot values and during counter-clockwise trajectory (B) in the inner (Foot-In) and outer foot (Foot-Out).** Each color corresponds to a non-linear range of %BW value featuring a significant p value at post-hoc analysis with respect to the corresponding anatomical region of the contralateral foot.

Figure 3 shows the vGRF (%BW) values in each of the eight anatomical regions of the left and right foot during LIN walking (A) and of Foot-In and Foot-Out during curved walking (B), averaged across subjects (Table 2). Each region is coloured according to the values of the mean vGRF. The colour code is reported in the calibration bar at the bottom of the figure.

**Table 2:** **Summary of the changes in vertical component of the ground reaction forces (vGRF) in the eight anatomical regions of the feet during linear and curved trajectories**

Region	vGRF (N)			vGRF %BW		
	Linear	Foot-in	Foot-out	Linear	Foot-in	Foot-out
**Hallux**	230.5^in^	176.6^a^	229.9	40.5^in^	31.0^a^	40.4
	*7.6*	*7.1*	*9.0*	*1.2*	*1.2*	*1.5*
**Lateral Toes**	125.1^in^	98.4	114.4	22.2^in^	17.4	20.2
	*6.6*	*6.1*	*6.6*	*1.2*	*1.1*	*1.2*
**I Metatarsal Head**	158.5	133.9^a^	161.6	27.8	23.3^a^	28.5
	*9.2*	*7.9*	*9.4*	*1.6*	*1.3*	*1.7*
**II-V Metatarsal Heads**	279.9^ou*t*^	285.6^a^	252.7	48.2^out^	49.5^a^	43.4
	*12.1*	*11.0*	*12.4*	*1.4*	*1.4*	*1.6*
**Medial Arch**	18.0	24.1	18.3	3.1	4.2	3.2
	*2.5*	*3.6*	*2.7*	*0.4*	*0.7*	*0.5*
**Lateral Arch**	89.3	109.1	102.2	15.7	19.0	18.0
	*6.6*	*6.5*	*6.8*	*1.2*	*1.0*	*1.2*
**Medial Heel**	239.5^out^	230.8^a^	177.5	42.0^out^	40.6^a^	31.1
	*9.0*	*7.7*	*7.6*	*1.4*	*1.3*	*1.1*
**Lateral Heel**	363.2^in^	298.9^a^	345.3	63.2^in^	51.8^a^	60.1
	*11.6*	*11.0*	*11.9*	*1.3*	*1.2*	*1.6*

Average values (± SE) from left and right feet. Foot-In and Foot-Out correspond respectively to the inner outer foot during curved walking. vGRF is represented as absolute values, normalized to body weight (%BW).

*out,* denotes a significant difference between linear foot and foot-Out (p<0.05). *in,* denotes a significant difference between linear foot and foot-In (p<0.05). *a,* denotes a significant difference between Foot-In and Foot-Out (p<0.05).

For LIN, the values of vGRF in each of the eight anatomical regions were distributed equally in both feet (Figure 3A). Accordingly, the values shown in Table 2 for each anatomical region are the averages for both feet. On the whole, the lateral part of the foot was loaded to a larger extent than the medial part, except for the hallux. This basic pattern was maintained in the three trajectories. However, modest (maximum 10%BW) but significant changes in vGRF in the anatomical regions of the foot sole were present between LIN and curved walking (see Table 2 and Figure 3B). For curved trajectories, Foot-In and Foot-Out were differently loaded with respect to LIN foot. Firstly, at heel strike, the lateral part of the Foot-In heel was unloaded with respect to that of LIN foot and Foot-Out. On the contrary, the medial part of the heel of Foot-Out was unloaded with respect to that of LIN foot and Foot-In. Regarding the medial and lateral arch, changes between the trajectories were negligible. Changes were instead again evident in the forefoot. At the level of the metatarsal heads, the lateral part of Foot-Out was unloaded with respect to that of LIN foot and Foot-In, while the medial part of Foot-In was unloaded with respect to Foot-Out. Strikingly different behaviour between the lateral and medial part of the foot was observed at the hallux and lateral toes. The hallux showed a clear-cut unloading in Foot-In with respect to LIN foot and Foot-Out, while the changes in the outer toes were smaller.

**Figure 4:**
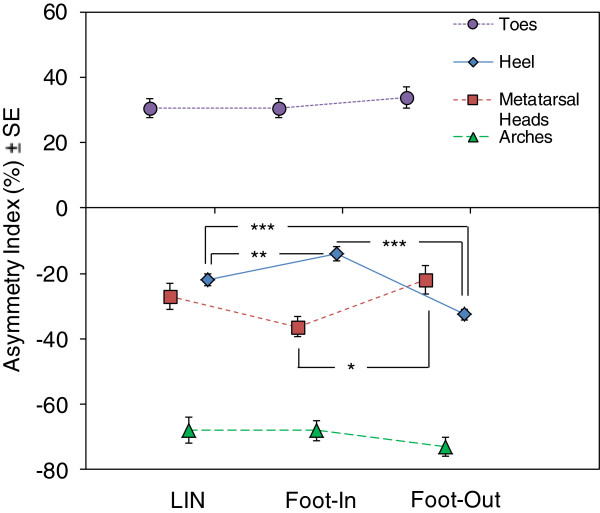
**Asymmetry index (AI) of the estimated ground reaction force distribution at the heel, metatarsal heads, arches and toes of the foot during linear (LIN) and curved trajectories (Foot-In, Foot-Out).** In the ordinate, larger negative values (average ± standard error, SE) of AI represent an increase in vGRF on the lateral part of the relevant foot region. During curved walking, AI of the Foot-Out heel became more negative than that of LIN foot and Foot-In. On the contrary, AI became less negative for Foot-In with respect to LIN foot. At the metatarsal heads, AI showed the opposite behaviour: for Foot-Out, it became slightly less negative with respect to LIN foot. On the contrary, for Foot-In, it became more negative than LIN foot and Foot-Out. *, p < 0.05; **, p < 0.005; ***, p < 0.0005.

In order to shortly describe the changes in vGRF distribution in the four medial and lateral anatomical regions of the foot during LIN and curved trajectories, we calculated an asymmetry index (AI) for each of the regions of LIN foot, Foot-In and Foot-Out, as:


Figure 4 shows that AI was negative for both linear and curved trajectories in the cases of the heel, arch and metatarsal heads, in keeping with the larger vGRF on the lateral than medial part of these foot regions. AI of the forefoot at the toes was positive, again regardless of the trajectory, indicating a larger vGRF on the hallux than lateral toes. Turning trajectory significantly modulated AI of the heel and the metatarsal heads, not so AI of the arch and toes. During LIN trajectory, AI of both heel and metatarsal heads showed an intermediate value between those for CW and CCW. One-way ANOVA showed a significant effect of feet (LIN foot, Foot-In, Foot-Out) on the heel (F(2,96) = 26.31; p < 0.0001), metatarsal heads (F(2,96) = 3.57; p < 0.05), but not on the arches (F(2,96) = 0.75; p = 0.47) and toes (F(2,96) = 0.37; p = 0.69). The post-hoc test showed that, during curved walking, AI of the Foot-Out heel became more negative than that of LIN foot (p < 0.0005) or Foot-In (p < 0.0005). This was in keeping with an increasing greater loading of the lateral part of the heel for Foot-Out with respect to Foot-In and LIN foot. On the contrary, AI became less negative for Foot-In with respect to LIN foot (p < 0.01), sign of a decreasing lateral loading of the heel. At the metatarsal heads, AI acted in the opposite way: for Foot-Out, it became slightly less negative (p = 0.62) with respect to LIN foot while, for Foot-In, it became more negative than LIN foot (p = 0.21) and Foot-Out (p < 0.05), in keeping with an increasing larger loading of the lateral part of the metatarsal heads of Foot-In with respect to Foot-Out and LIN foot.

### Trunk inclination during linear and curved walking

**Figure 5:**
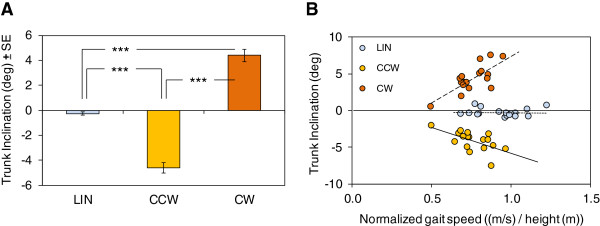
**Trunk inclination during linear and curved trajectories and its relation to walking speed. A**. Trunk inclination during the linear (LIN) and curved trajectories (counter-clockwise, CCW; clockwise, CW). The curved trajectories present a similar but opposite value. **B**. Relationship between walking speed normalized to height and trunk inclination across subjects. A linear relationship is present between inclination and speed but only during curved trajectories. *, p < 0.05; **, p < 0.005; ***, p < 0.0005.

Trunk inclination in the frontal plane was dependent on the trajectory (Figure 5A). During LIN walking, inclination ranged between −1 and +0.9 deg. For curved paths, the trunk was inclined toward the interior of the trajectory: for CCW, it was inclined to the left (negative values), ranging across subjects between −7.6 and −2.1 deg, while for CW it was inclined to the right (positive values), ranging between +0.5 and +7.6 deg. On average, trunk inclination was −0.3 deg ± 0.1, −4.6 deg ± 0.4, and +4.4 deg ± 0.5 during LIN, CCW and CW, respectively. ANOVA showed a significant effect of the trajectories on inclination (F(2,30) = 162.3; p < 0.0001). Inclination values differed from each other for the three trajectories (post-hoc, p < 0.0001).

Figure 5B shows that, across subjects, even moderate changes in walking speed had an effect on trunk inclination, i.e. the faster the gait speed (normalized to height), the more inclined the trunk, both for CW (y = 12.63× – 5.28, p < 0.01, r^2^ = 0.56) and for CCW (y = −7.03× + 1.2, p < 0.01, r^2^ = 0.36). Clearly, no relationship was found between speed and trunk inclination during LIN trajectories (y = −0.18 – 0.12×, p = 0.90, r^2^ = 0.001).

**Figure 6:**
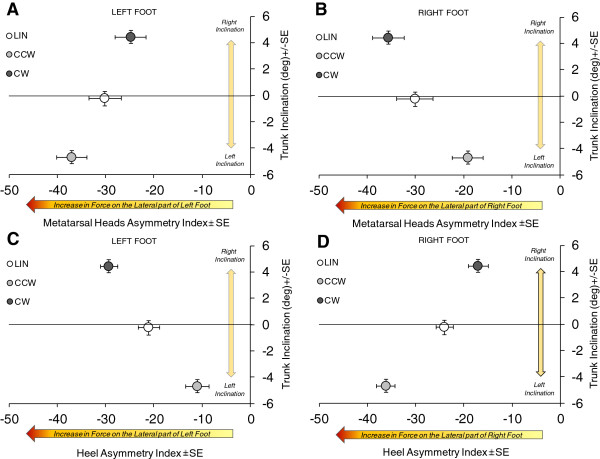
**Dependence of the asymmetry index (AI) of the vGRF distribution at the metatarsal heads of the left and right foot (respectively, A and B) and at the heel of the left and right foot (respectively, C and D) on trunk inclination during linear (LIN), counter-clockwise (CCW) and clockwise (CW) trajectories.** In the abscissa, larger negative values (average ± standard error, SE) of AI represent an increase in vGRF on the lateral part of the relevant foot region. In the ordinate, positive or negative values (average ± SE) represent respectively inclination towards right or left side. See text for explanation.

As expected, the asymmetry index (AI) of the vGRF values of each foot exhibited a clear-cut dependence on trunk inclination during the CW and CCW trajectories. This dependence was opposite for the back (heel) and for the front part (metatarsal heads) of both feet. In Figure 6, the mean trunk inclination (right, positive values) is plotted against the mean AI (abscissa), for the LIN, CW and CCW trajectories. Briefly, during CW, the heel of the right foot (Foot-In, panel D) was loaded relatively more in its medial part, while during CCW, the heel of the same foot (now Foot-Out) was loaded more in its lateral part. A mirror pattern was observed for the front part of the right foot (panel B). In this case, during CW the metatarsal heads of the right foot (Foot-In) were loaded more in their lateral part, while during CCW the metatarsal heads of the right foot (now Foot-Out) were loaded more in their medial part (right bottom panel). Panels A and C show similar but opposite patterns for the left foot.

## Discussion

Several studies have shown that there is a moderate reduction in walking speed along a curved path with respect to walking in a straight line [4, 10, 19, 30–37], dependent on the angle of turn [38]. This reduction may be due to biomechanical constraints, to the necessary tuning of the locomotor command directed to the limbs [5], and/or to the need to stabilize the head or the lower limb joints [30, 39]. Curved walking also involves different neural processes compared to straight-path walking. Cognitive flexibility and set-shifting processes uniquely contribute to how individuals navigate curved paths [40, 41].

The present findings confirm the slight decrease in velocity during curved compared to linear walking in young healthy subjects [19]; at the same time, they increase our insight into the mechanisms underlying curved walking, showing an interaction in the pattern of the vertical component of the ground reaction force (vGRF) within and between the two feet during curved walking. While the insole output of the two feet is symmetric during linear walking, the pattern diverges during curved walking. Further, the modifications of the vGRF from the linear pattern are differently distributed in the various parts of the feet, depending on the position of the foot with respect to the orientation of the curved path and on the time-course of the stance phase.

In this study, both CW and CCW trajectories have been performed and have been separately examined as different conditions, instead of collapsing CW and CCW walking into one ‘turning’ condition. This was made in order to exclude that a ‘preferred’ leg would have produced different results when matched to a ‘preferred’ direction, with potential differences in walking velocity for CW and CCW turning, since leg preference might contribute to balance asymmetries and be associated with different foot placements [42, 43].

### Profiles of the ground reaction force during the stance phase

The vGRF time profiles during the stance phase of gait are characterized by two peaks [44]: the first peak corresponds to the heel strike and weight-acceptance phase, the second peak to the last part of the stance until toe off, when the vGRF becomes zero. With respect to the straight path, the force profiles of both feet during curved walking show a decrease of the first and last peaks and an increase of force during the trough corresponding to mid-stance. Further, when compared between feet during curved walking, the peaks are slightly but significantly lower, and the trough slightly but significantly higher, in Foot-In than Foot-Out (see Figure 1B). Therefore, the outer foot would play a predominant role in steering the body, as shown by the higher value of the forces during both the first and second peak. During mid-stance, however, the higher force at the inner foot is likely due to the body pivoting on it during turning. Such a pattern of vertical forces during stance had been observed previously, as well as its dependence on curvature and velocity [6, 45].

The smaller amplitude of the first and last peaks (both feet) in curved with respect to linear walking may be in part related to the fact that the insole records only the vertical component of the force. This depends on the distance between the instantaneous positions of the center of mass (CoM) of the body and of the vGRF of the stance foot. In this study, we did not compute the position of the CoM of the body. However, its distance from the vGRF must have increased during curved with respect to straight walking, as clearly shown by the inclinometer measurements and by the strong relationship between trunk inclination angle and velocity of curved walking (see Figure 5B) [4]. Therefore, the diminished values of vGRF must reflect the new vertical force equal to F = F * cos inclination-angle. The inter-peak trough during curved trajectories was however increased with respect to linear walking. This must depend on the overall different distributions of the vGRF during the stance phase in curved with respect to linear trajectories. The increase of the trough would be even larger were it not for the effect of the inclination. This increase must be related to the production of the curved trajectory, whereby the body pivots in the horizontal plane on the foot arch during the yaw rotation, however briefly, for the production of a small angular deviation for each step [4], rather than rolling onto the forefoot along the para-sagittal plane. Of note, the stance duration is normally slightly longer for the inner than the outer foot (see Table 1) [9]. The increased load of the mid-foot during mid-stance is common to both the inner and outer foot, so that each foot (albeit more so the inner foot) can contribute a compliant support for the body weight [46] step after step, as one walks a curved path.

### Trunk inclination

The generation of the centripetal force at foot level can be effective only if trunk control is adequate. This must requires a delicate coordination pattern of muscle activities along the body. That this is so has been shown some time ago by Courtine et al. [7] and Orendurff et al. [47], who found that amplitude and timing characteristics of limb and trunk muscle activities were significantly correlated to the spatial and temporal gait adaptations associated with curvilinear locomotion. Ultimately, the accurate and appropriate position of the inner and outer foot should be the result of the coordinated spatial and temporal modulation of muscle activities of the entire kinematic chain. This is tuned as a function of both spatial and temporal features of gait [7] and assures the critical control of upper body stability [48].

Some authors already used these vertical force variables as a measure of balance during walking [41, 49]. In the present study, a relationship between trunk inclination and speed normalized to height was clearly present. During curved as opposed to linear walking, the body mid-point comes closer to the inner and more distant from the outer foot during the respective stance phases. The tighter the curve, the more the body mid-point shifts towards the inner foot during its stance phase until the body mid-point bypasses the inner foot toward the centre of curvature [9, 6].

In addition to the variability across subjects of body kinematics and asymmetry indexes, other spatial features of gait may play a role in the relatively large scatter of the relationship between trunk inclination and gait speed shown in Figure 5B. These features have not been directly measured in this study. However, they might include the yaw angle of the feet and the step width [4], which may not be exactly the same in all subjects.

### Spatial distribution of the ground reaction force underneath the foot sole

**Figure 7:**
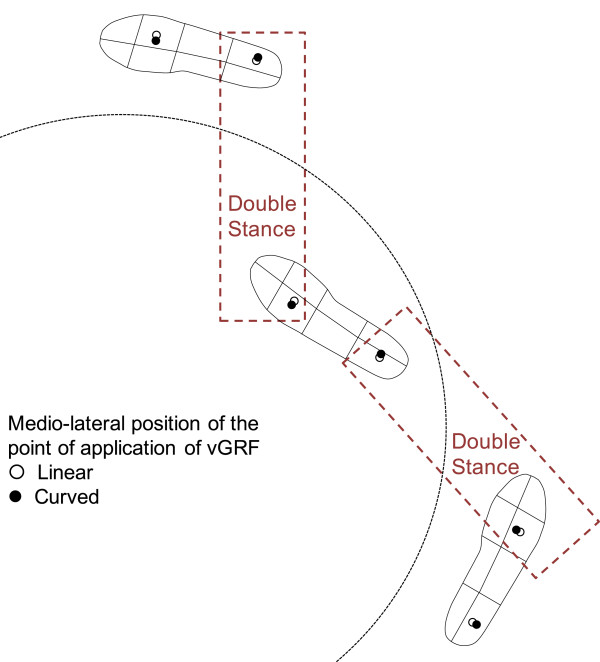
**Description through the asymmetry index (AI), of the shifts of vGRF subserving the controlled production of the centripetal force during curved walking.** Black points indicate the AI during counter-clockwise trajectories; the white points indicate AI during linear trajectories. Note in the Foot-Out the displacement from medial to lateral position of vGRF at the heel and from lateral to medial position of vGRF at the metatarsal heads with respect to linear walking. The reverse occurs in the Foot-In.

The subdivision of the foot print into eight regions (four for each longitudinal half) allowed to further explore the mechanisms subserving the controlled production of centripetal force during curved walking. For simplicity, we discuss here in detail only the most salient features of vGRF during the load-acceptance phase and the late stance phase (see Figure 7). Undoubtedly, the measurement of vGRF *per se* under the feet cannot provide the generation of centripetal force, which is a horizontal force that is not measured by the insoles. However, the instantaneous position of the point of application of this vertical force during turning, and its displacement with respect to what happens during linear walking, can give insight in the production of the gap between the center of mass and this application point. This distance created between center of mass and center of pressure in the medio-lateral plane produces a disequilibrium torque driven by gravity that is responsible for accelerating the body toward the center of the trajectory, very much as a similar torque during linear walking produces a disequilibrium in the sagittal plane that accelerates the body forward [50, 51].

At heel strike, compared to linear walking, CCW is associated with displacement of the point of application of the vGRF toward the lateral part of the heel (as indicated by the increased AI) of the right (*outer)* foot. This outer displacement produces an increased medio-lateral distance between the center of mass of the body (displaced toward the interior of the trajectory) and the application point, therefore a *larger* torque acting in the frontal plane and pushing the center of mass toward the inner part of the trajectory, favoring centripetal acceleration [52]. Then, once the body rolls over the foot and the vGRF moves to the metatarsal heads, the force exhibits a moderate displacement toward the medial part of the forefoot. This relatively *reduces* the torque acting on the body directed to the inner part of the trajectory. Overall, it seems that a *push-pull mechanism* operates on the body mass: when touching the ground, the outer foot helps the body ‘fall’ toward the interior of the trajectory, while in the late stance phase its front part brakes any further inclination of the body to the interior of the trajectory by having its medial (*inner*) part put pressure on the ground.

Under the same CCW condition, compared to linear walking, the vGRF of the left (*inner*) foot at foot contact is shifted toward the medial part of the heel. This increases the net torque acting on the body mass directed to the interior of the trajectory. The body fall to the interior is therefore being favored by the inner foot during the weight acceptance phase. On the other hand, the small displacement of the point of application of the vGRF at the lateral metatarsal heads during the evolution of the stance phase of the inner foot (AI is increased) brakes any further fall toward the interior of the trajectory, thereby favoring the movement of center of mass toward the exterior of the trajectory.

The described features are closely mirrored in CW walking (Figure 6). Overall, it seems that *a double push-pull mechanism* operates on the body mass. At heel contact and weight acceptance, both feet create a torque pushing the body to the interior of the trajectory. On the contrary, both forefeet have an opposing action, or an action braking the inward fall, by increasing the torque directed to the outer part of the trajectory. During curved walking, the late stance phase has a braking action, promptly counteracted by the heel contact of the opposite foot when the double stance phase supervenes. The double-stance phase reinstates the appropriate inward fall due to the shift of the vGRF point of application to the lateral part of the outer foot heel when it touches the ground.

Thus the feet behave *functionally* in the same way, regardless of their being the inner or outer foot. This is possible because of the asymmetry of the path of the vGRF below the feet: from medial (at heel strike) to lateral (at toe off) for the inner foot, and from lateral (at heel strike) to medial (at toe off) for the outer foot. Obviously, the net effect of this push-pull pattern cannot be but an inward thrust, as witnessed by the trunk inclination toward the interior of the trajectory, which counteracts the centrifugal force during steady-state turning.

### Limitations

Precise calculation of the torques presupposes a complete quantitative description of the generation of the centripetal force. We did not record the yaw placement of the feet (inner and outer) with respect to the direction walked. Foot distance from the trajectory can have major impact on the vGRF distribution and the direction of the ensuing torques [53]. For instance, patients affected by different ailments may exhibit different foot placements from normal subjects [11, 14, 54] and this can affect their capacity to smoothly walk a circular path. Even in our normal subjects here, inconsistent foot placement may have been the source of the observed non-negligible variability in the recorded variables [55]. Another limitation is that we did not record the body segment kinematics or compute the body’s center of mass. Measuring the medio-lateral trunk inclination can provide easy, comprehensive and meaningful information about gait pattern during curved paths. However, from the mere measure of body inclination, one can have only indirect information on the biomechanics of curved walking. Data on foot placement and center of mass position together would have allowed a precise estimation of the torques accompanying circular walking.

Further, by necessity, plantar insoles, as compared to force platforms, give information on the vertical component of the force produced by the walking body, and cannot reveal the size of the shear forces [56]. Devices able to yield continuous measures of plantar shear-forces during walking are not readily available. Shear forces inevitably have a non-negligible value [57], the more so during curved walking because of the trunk inclination accompanying this locomotor task, and do certainly play a role in the production of mechanical effects in the frontal plane [47, 13].

Therefore, the present results can give insight into the way foot placement exploits gravity in order to produce the body inclination during curved walking, by creating the properly oriented medio-lateral torques. The effect of the shear forces in favouring or braking the inward fall cannot be addressed here. However, we would consider that shear forces are more the consequence than the cause of the body inclination, and may not be a continuously controlled variable in the production of curved walking on solid, non-slippery ground.

## Conclusions

The present findings provide comprehensive and meaningful information about the pattern of GRF in curved walking in healthy subjects and give hints about the role of trunk inclination in accomplishing this task. In spite of the limitations mentioned above, the combined use of plantar insoles and accelerometer systems is an easy and relatively low-cost method. The advantage of the insoles is that there is no constraint on foot placement and that many consecutive strides can be recorded, while knowledge about the medio-lateral trunk inclination when walking along curved pathways may be useful in addressing problems in the control of equilibrium during turning.

The hypotheses put forward in this study should be tested in patients with problems in turning while walking. We predict that in these patients both distribution of plantar pressures as measured by insoles and of trunk inclination as measured by the accelerometer would be abnormal. We would also predict that the largest deviations from normal behaviour should be more evident during curved than linear trajectories, in which balance control is less critical [40].

## Abbreviations

vGRF: Vertical ground reaction force
BW: Body weight
SE: Standard error
LIN: Linear
CW: Clockwise
CCW: Counter-clockwise
%GC: Percentage of total gait cycle duration
AI: Asymmetry index
HC: Heel contact
MS: Mid-stance
TO: Toe off.
